# Top-cited articles on vital pulp therapy for irreversible pulpitis in permanent teeth: a bibliometric analysis

**DOI:** 10.1007/s10266-026-01327-1

**Published:** 2026-01-28

**Authors:** İkbal Sena Çelebi Keskin, Ferda Karabay

**Affiliations:** 1 Department of Endodontics, Faculty of Dentistry, Istanbul Medeniyet University, Orhanlı Campus, 34956 Tuzla, Istanbul, Turkey; 2 Department of Restorative Dentistry, Faculty of Dentistry, Istanbul Medeniyet University, Istanbul, Turkey

**Keywords:** Bibliometric analysis, Citation trends, Irreversible pulpitis, Pulpotomy, Vital pulp therapy

## Abstract

Vital pulp therapies (VPTs) have emerged as a potential alternative to nonsurgical root canal treatment (RCT) for irreversible pulpitis (IP) in permanent teeth. Although many studies explored VPT efficacy, no bibliometric analysis has focused on this topic. A systematic Web of Science search (May 29, 2025) identified 319 eligible articles using keywords related to VPTs and IP. Only full-length articles published in English and indexed within the Dentistry, Oral Surgery, and Medicine categories were included, while dissertations, conference papers, editorials, letters, retracted publications, and articles with unspecified document types were excluded. The 50 most-cited papers were analyzed for citations, authorship, institutions, countries, keywords, and study design. Data were processed with SPSS and VOSviewer. The top 50 most-cited articles accumulated 3,869 citations. The most-cited article (356 citations) was the European Society of Endodontology’s position statement (2019). A significant increase in publications occurred after 2015, with peak outputs in 2019 and 2022. Most studies were clinical trials, mainly randomized, followed by non-randomized and observational studies. Iran, the USA, Jordan, India, and Ireland were leading contributor countries, with Shahid Beheshti University, Jordan University of Science and Technology, and Dublin Dental University Hospital standing out as key institutions. Author analysis revealed that Asgary S, Taha NA, and Duncan HF were among the most influential researchers. Keyword mapping showed “pulpotomy” as the dominant focus, followed by “pulpitis” and “irreversible pulpitis.” There is growing scientific interest in VPTs for IP, though more high-level evidence is needed. This analysis identifies current research patterns and gaps to guide future investigations.

**Supplementary Information:**

The online version contains supplementary material available at https://doi.org/10.1007/s10266-026-01327-1.

## Introduction

Pulpitis is a clinical and histological term referring to inflammation of the dental pulp, classified clinically as reversible or irreversible and histologically as acute, chronic, or hyperplastic according to the American Association of Endodontists (AAE) Glossary of Endodontic Terms, updated in 2020 [1]. Irreversible pulpitis (IP) has been clinically categorized into two subtypes: asymptomatic IP and symptomatic IP [1]. Asymptomatic IP is characterized by the absence of clinical symptoms; however, inflammation resulting from caries, caries excavation, or trauma is present. In contrast, symptomatic IP is defined as a clinical condition characterized by prolonged thermal pain, spontaneous pain, and referred pain [1]. For both types of IP, it is indicated that the vital inflamed pulp has no capacity for healing [1], and nonsurgical root canal treatment (RCT) is recommended as the standard therapeutic approach for permanent teeth [2]. However, in recent years, vital pulp therapies (VPTs) aimed at preserving dental pulp vitality, particularly pulpotomy, have emerged as a promising alternative for teeth diagnosed with IP [3–7]. In the Position Statement on Vital Pulp Therapy published in 2021, the AAE emphasizes that pulpectomy is not mandatory in every case of IP and highlights that more conservative treatment options should also be evaluated [8]. Similarly, the European Society of Endodontology (ESE), in its guidelines published in 2023, recommended that RCT or full pulpotomy are appropriate treatment options for cases of pulpitis associated with spontaneous pain in permanent teeth [9]. However, AAE generally recommends complete caries removal to properly assess the pulp and make clear treatment decisions, whereas ESE takes a more conservative approach in deep caries and suggests selective or stepwise removal to reduce the risk of pulp exposure. When the clinical signs indicate irreversible pulpitis, neither organization recommends selective caries removal. In such cases, both guide the clinician toward the appropriate vital pulp therapy or root canal treatment [8, 9].

VPTs are minimally invasive procedures aimed at preserving and maintaining pulp vitality by covering the pulp tissue with a biocompatible material [2]. These procedures include indirect and direct pulp capping, partial pulpotomy, and full pulpotomy [1]. Indirect pulp capping involves the placement of a biocompatible material close to the pulp tissue without exposure, whereas direct pulp capping entails applying a biocompatible material directly onto the exposed pulp tissue [10]. Partial pulpotomy involves the removal of only the superficially infected pulp tissue, whereas full pulpotomy entails the complete removal of the coronal portion of the pulp followed by the application of a biocompatible material [1]. The success of VPTs depends on various parameters, including the type and properties of the materials utilized [5, 11], the specific clinical techniques employed during the procedure [12], and the pulpal condition [13].

Although VPT is an old concept, its use in teeth with pulp exposure remained limited for many years because the outcomes were often unpredictable [14]. Even though research showed that the pulp could heal, the inconsistent success rates prevented VPT from being widely accepted as a true alternative to root canal treatment [14, 15]. Over the last two decades, however, the introduction of bioactive hydraulic calcium silicate cements, improved understanding of pulp biology, and advances in tissue management have renewed interest in VPT and led to markedly better clinical outcomes [14, 16–18]. In recent years, the increasing number of studies in the literature on VPTs for teeth with IP has provided various data regarding the effectiveness and clinical applications of this method [19, 20]. Understanding the effectiveness of VPTs in teeth with IP is of great importance for the development of future clinical protocols and the promotion of more minimally invasive approaches [21].

Bibliometric analysis is an effective method for examining large-scale unstructured data through systematic, objective, and replicable techniques to uncover and visualize the scientific literature development and evolutionary dynamics of a specific discipline [22]. It helps researchers analyze existing knowledge, identify gaps, generate new ideas, and position their work within the broader context of the field [23, 24]. Unlike systematic or scoping reviews, which evaluate the quality and outcomes of studies, bibliometric analysis focuses on publication trends, citation patterns, co-occurrence analyses, collaboration networks, and how research in the field changes over time [25, 26]. Citation analysis is regarded as a primary bibliometric method. Citation analysis helps identify the seminal works guiding the development of a field, prominent researchers, contributing institutions, and the most influential journals [27]. In the field of endodontics, citation analysis studies have been conducted on various topics such as regenerative endodontics [28, 29], photodynamic therapy [30], endodontic microbiology [31], silicate-based endodontic materials [32, 33], VPTs [34, 35], micro-CT applications in endodontics [36], and laser applications [37, 38].

To the best of our knowledge, no bibliometric analysis has been published on VPTs in permanent teeth with IP, despite the existence of various systematic reviews addressing different aspects of this topic [20, 21]. However, these reviews mainly focus on clinical outcomes and material-related factors and therefore do not reflect how the research activity in this field has evolved over time. For this reason, a bibliometric analysis is needed to illustrate publication trends, highlight influential authors and institutions, and clarify how key themes in VPT research have developed. In this study, a bibliometric analysis of VPTs in teeth with IP was conducted. The existing trends in the literature, author collaborations, key concepts, and citation dynamics were evaluated to clarify how the evidence base has developed, to identify the main contributors in the field, and to outline the thematic structure of VPT research. By presenting an overview of the current state of research in this field, the study aimed to provide a broader scientific context and guide future research directions.

## Materials and methods

In this study, since the data were obtained from previously published studies, an institutional ethics committee approval was not sought.

### Data collection process

For the bibliometric analysis of VPTs in permanent teeth with IP, the search terms listed in Table S1 were first determined. To ensure thorough identification of all relevant articles, the keywords were carefully selected. In this bibliometric analysis, both mature and immature permanent teeth were included without differentiation based on root development, while primary teeth were excluded. The first term—related to permanent dentition—and the second term—related to VPTs performed in patients diagnosed with IP—were constructed by combining the most frequently used free terms and Medical Subject Headings (MeSH) terms. As the third term, the keywords related to IP were created by combining the most frequently used free terms and the terms used to describe IP in the Glossary of Endodontic Terms [1].

To identify articles published since 1975, an electronic search was conducted in the Web of Science (WoS) database (http://www.webofknowledge.com) on May 29, 2025. All searches were conducted using the 'All Databases' option in WoS. The initial search, conducted using the first set of search terms, yielded 152,292 articles. With the addition of the second set of search terms, the number of articles was reduced to 6,844. The inclusion of the third set of search terms further narrowed the results to 3,689 articles. When all three sets of terms were combined and the Preprint Citation Index was excluded from the results, a total of 436 articles were identified. This dataset was then further refined based on predefined inclusion and exclusion criteria.

- Restricting the results to articles published in English resulted in 420 articles.
- Filtering for studies in the fields of dentistry, oral surgery, and medicine reduced the count to 340 articles.
- Finally, after excluding dissertations, editorial materials, conference proceedings, retracted publications, letters, and articles with unspecified types, 319 articles remained. All filtered studies were exported in full record format into Microsoft Excel (Redmond, WA) and organized for further analysis.

### Article selection and citation analysis

To determine the top 50 most-cited articles [27, 31], two independent researchers first examined the titles and abstracts of the 319 articles ranked by citation count in the WoS database. If a decision could not be made solely based on the title and abstract, the full text was reviewed. Apart from the predefined exclusion criteria, the eligibility criteria focused on studies where the entire study or a significant portion was related to VPTs in permanent teeth with IP or had a relevant impact in this field. The reviewers compared their findings, and any discrepancies were resolved through discussion and a consensus-based decision, which included reviewing the full text of the articles and reassessing the relevant literature. Inter-examiner consistency was assessed using Cohen’s kappa coefficient (0.85).

Once the top 50 most-cited articles were finalized, a more comprehensive analysis was conducted by manually retrieving their citation counts from Scopus on May 30, 2025. Considering that citation counts may vary over time, all data were collected on the same day to maintain consistency. Based on citation counts from the WoS database, the top 50 most-cited articles were ranked from highest to lowest and presented in Table S2**,** incorporating the results from all the searched databases. In articles with the same number of citations, the more recently published one was positioned higher in the ranking.

For each of the top 50 most-cited articles, the following variables were recorded:

- Article title.
- Year of publication.
- Journal name and impact factor (Journal Impact Factor—JIF 2024).
- First author's name.
- First author's affiliated institution.
- First author's country.
- Institutional collaboration.
- Keywords.
- Methodological design.

Institutional information was defined based on the first author’s affiliation address; if the author was affiliated with multiple institutions, each was considered separately. In cases where more than one first author was indicated in a study, the names of both authors, along with their affiliated institutions and countries, were included in the dataset. For the classification of institutional collaboration types, the institutional affiliations and address information of co-authors were considered without reference to departments. Publications in which all contributing authors were affiliated with the same research institution, irrespective of departmental differences, were categorized as ‘individual institution’ collaborations. Studies involving more than one institution within the same country were defined as ‘multi-institutional’ collaborations, whereas those including institutions from different countries were classified as ‘international’ collaborations. To assess the journal's impact, the most recent (2024) Journal Impact Factor, as listed by Clarivate's Journal Citation Reports, was taken into consideration. The studies were categorized based on the research methodology they employed. These categories included randomized and non-randomized clinical trials, observational studies, systematic reviews, meta-analyses, review articles, basic science studies, guidelines, and umbrella reviews.

The data were recorded using Excel software. The collected data were analyzed and interpreted using appropriate descriptive statistical methods using SPSS version 21 (IBM Corporation, USA). The co-authorship network and the conceptual relationships between keywords were analyzed and visualized using VOSviewer (version 1.6.20) software.

## Results

The top 50 most cited articles published in the field of VPTs in permanent teeth with IP are presented in Table S2 in descending order according to the number of citations. The most cited article received 356 citations and was published in 2019 in the International Endodontic Journal (IEJ) by Duncan et al. (Table S2). This publication represents the official position statement of the European Society of Endodontology, titled 'Management of Deep Caries and the Exposed Pulp (Table S2). The article with the lowest number of citations received 27 citations. The total number of citations received by the 50 most cited articles was 3869, and the average number of citations per article was calculated as 77.38. Among the most highly cited articles, 11 studies have received 100 or more citations and are considered “citation classics” in the literature.

### Journal and year of publication

**Fig. 1:**
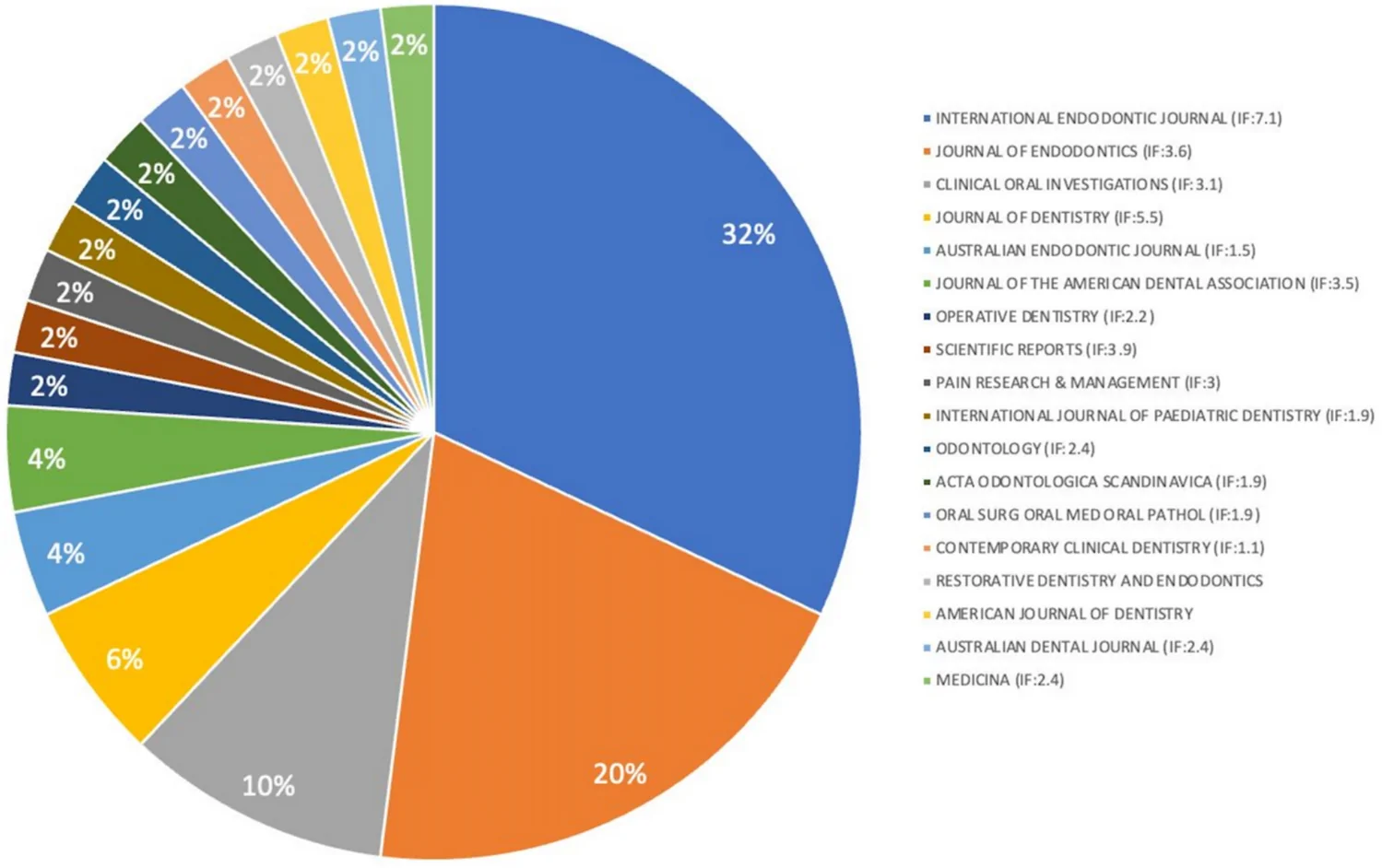
Percentage distribution of journals publishing the top 50 most-cited articles (with 2024 journal impact factors)

The top 50 most cited articles on VPTs applied in the treatment of IP in permanent teeth were published across 18 different journals, and all were written in English. Among the 18 journals examined, it was determined that 12 had published only a single article (Fig. 1). In addition, two journals had published two articles each, while the remaining four journals had published three or more articles (Fig. 1). The impact factors of these four journals range between 3.1 and 7.1 (Fig. 1). An examination of the journals in which the most cited articles were published shows that the IEJ had the highest share with 32%, followed by the Journal of Endodontics (JOE) with 20% and Clinical Oral Investigations with 10% (Fig. 1). These three journals collectively published 62% of the top-cited articles in endodontics.

**Fig. 2:**
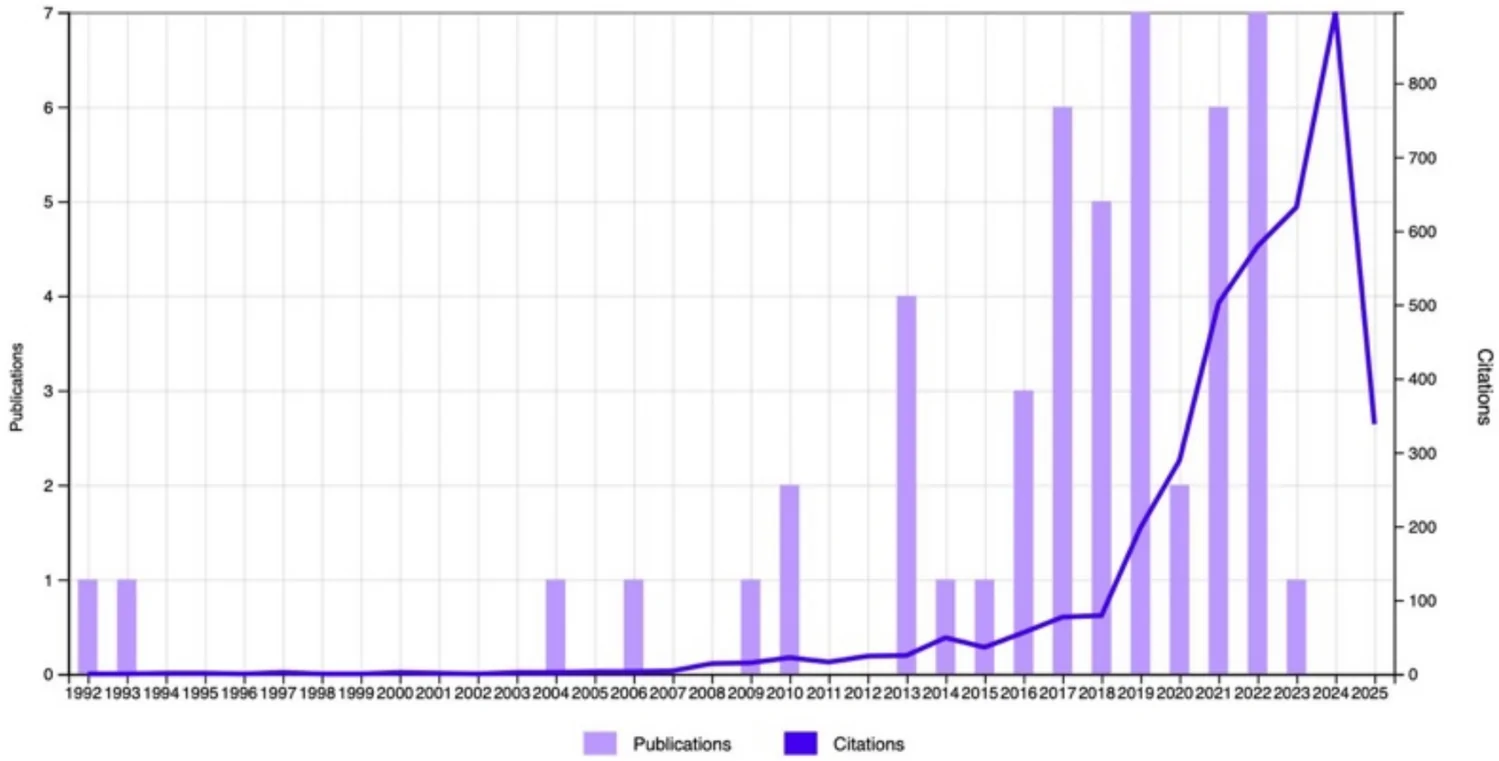
Temporal trends in citation counts of the top 50 most-cited articles

The publication dates of the top 50 most cited articles range from 1992 to 2023 (Fig. 2). The years with the highest number of publications were 2019 and 2022, each contributing seven articles to the top-cited list (Fig. 2). Notably, only five of these highly cited studies were published before 2010, while a marked increase in publication frequency has been observed after 2015 (Fig. 2).

### Authors, countries, and institutions of origin

**Fig. 3:**
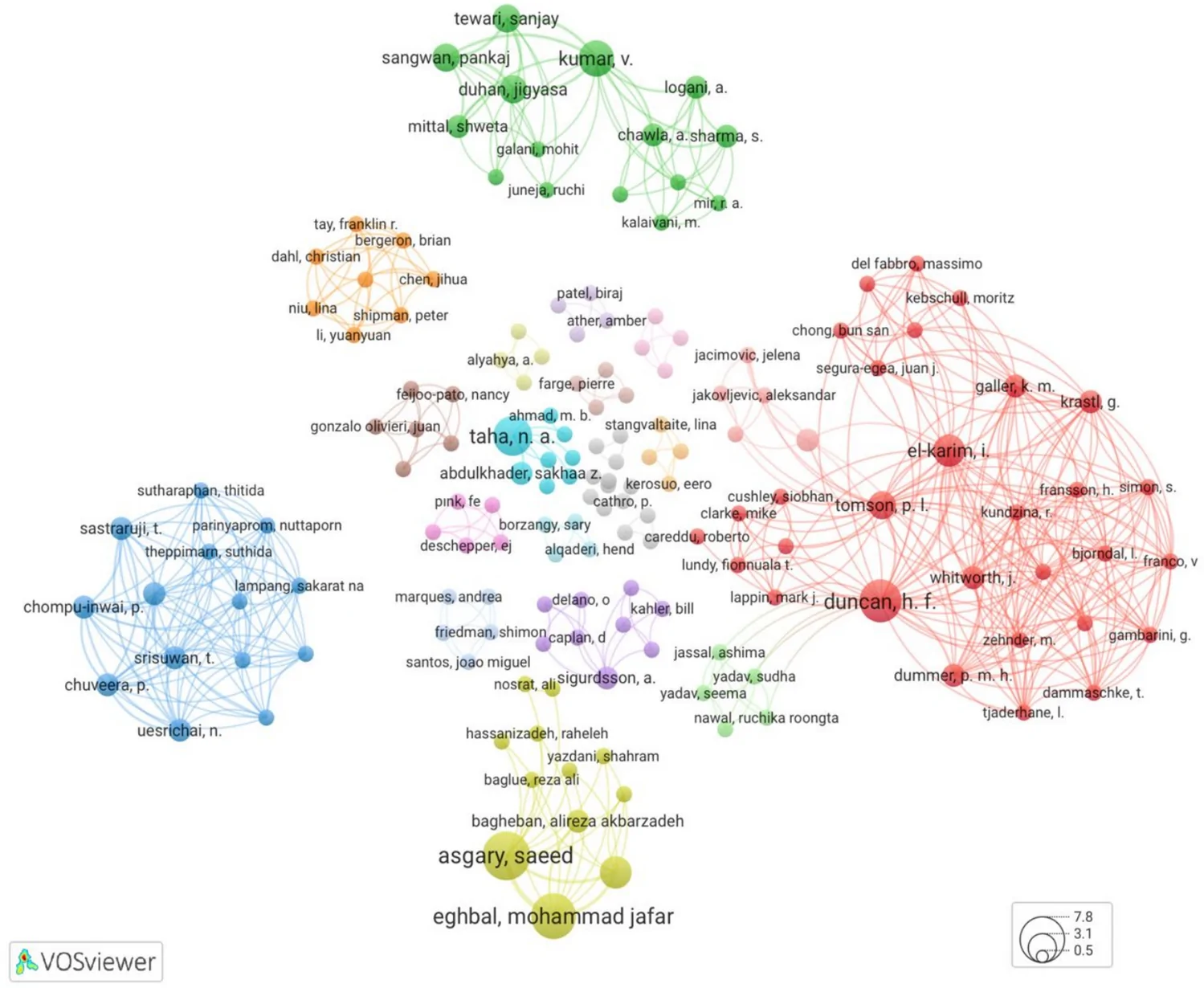
Co-authorship network map of the authors contributing to the top 50 most-cited articles. From VOSviewer interface; in analysis option “Association strength” selected as normalization method and in the visualization settings, the ‘’Weights’’ option was set to ‘’Documents’’, so that author label sizes reflect the number of publications contributed by each author. The minimum cluster size was set to 1

Examination of the top 50 most-cited articles showed that a total of 160 unique authors contributed to these studies (Table S2). Among the 50 articles analyzed, 2 were written by a single author, 10 were authored by two authors, and the remaining 38 included the participation of three or more authors (Table S2). A total of 37 researchers served as first authors in the 50 most-cited articles (Table S2). There is only one article in which two researchers are listed as first authors (Table S2). The analysis revealed that the most-cited author was Asgary S, with 7 articles and a total of 600 citations (Table 1). Asgary S was followed by Taha NA with 6 articles and 556 citations, Duncan HF with 4 articles and 624 citations, and Eghbal MJ with 2 articles and 126 citations (Table 1). When evaluated in terms of total author contributions, Asgary S ranked first with 9 publications and 713 citations (Fig. 3). He was followed by Eghbal MJ with 8 publications and 640 citations, Duncan HF with 7 publications and 829 citations, Taha NA with 6 publications and 508 citations, and Kumar V with 5 publications and 215 citations (Fig. 3).

**Table 1:** First authors with two or more top-cited publications

First author	No. of articles	No. of citations*	Citations per article*	H index*
Asgary, S	7	600	85,71	33
Taha, NA	6	556	92,67	17
Duncan, HF	4	624	156	31
Eghbal, MJ	2	126	63	26

**Fig. 4:**
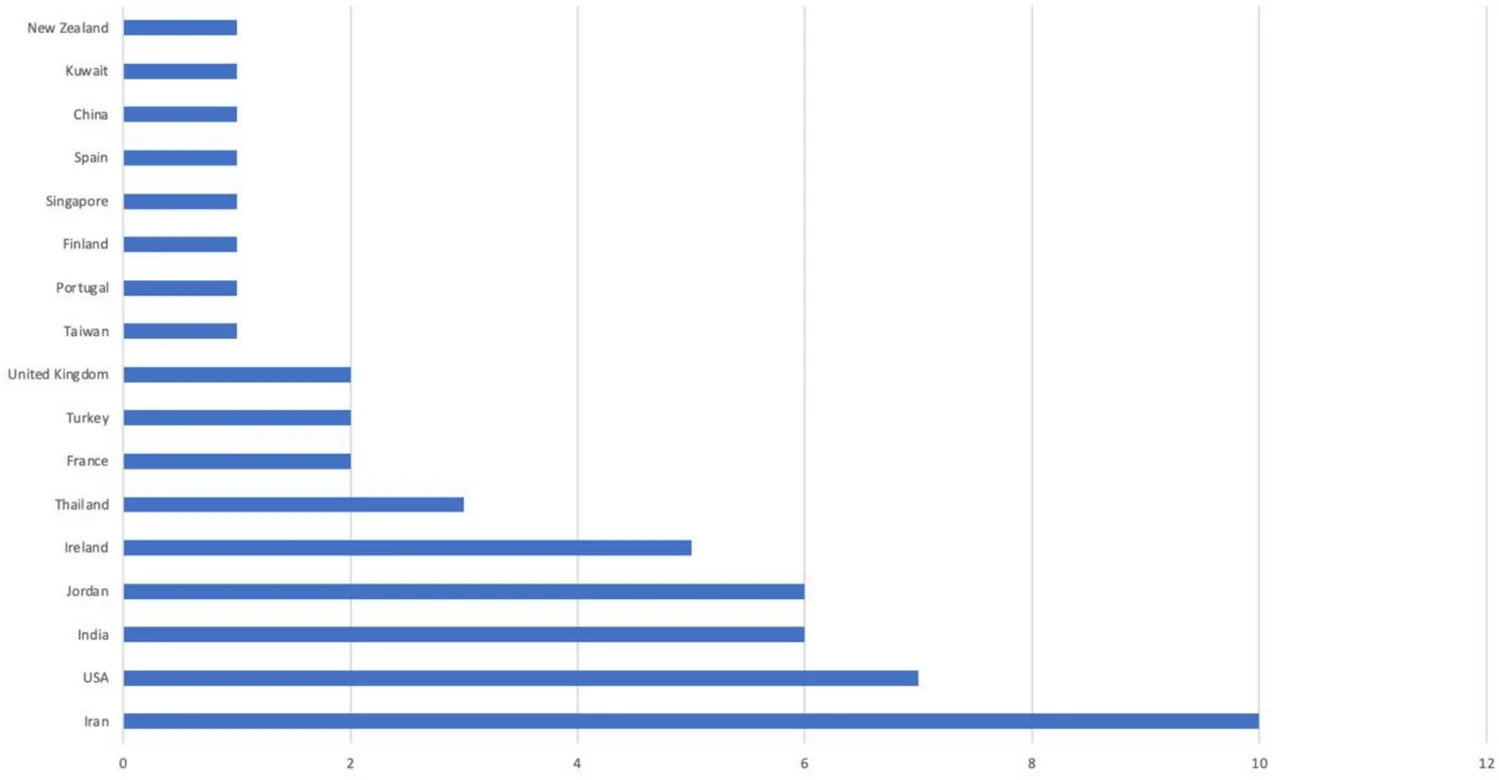
Countries of origin of the top 50 top-cited articles

The country and institution of origin were identified based on the affiliation address of the first author. Based on the analysis, the 50 most-cited articles were published by authors from 17 different countries (Fig. 4). Iran, with 10 articles, was the country with the highest number of publications, followed by the USA with 7 articles, India and Jordan with 6 articles each, and Ireland with 5 articles (Fig. 4).

A total of 30 institutions contributed to the top 50 most-cited publications; additionally, in 2 of the articles, the authors' affiliation was listed as ‘private practice’. Institutions with two or more publications are listed in Table 2. The institution with the highest number of publications was Shahid Beheshti University, with 9 articles and 726 citations (Table 2). It was followed by Jordan University of Science and Technology with 6 articles and 556 citations, and Dublin Dental University Hospital with 5 articles and 679 citations (Table 2).

**Table 2:** Institutions with two or more top-cited articles

Institutions	No. of articles	No. of citations*	Citations per year*
Shahid Beheshti University	9	726	59,34
Jordan University of Science and Technology	6	556	75,04
Dublin Dental University Hospital	5	679	114,08
Post Graduate Institute of Dental Sciences, Rohtak	3	171	23,5
Chiang Mai University	2	171	23
All India Institute of Medical Sciences, New Delhi	2	70	12,77

*for WoS

Among the top 50 most-cited articles, collaboration analysis revealed that 24 studies were conducted within individual institutions, while 13 involved multi-institutional collaborations and 13 were produced through international collaborations (Table 3). Internationally collaborative articles most frequently involved institutions from the United Kingdom (*n* = 5), followed by Ireland (*n* = 4). Collaborations with institutions in the United States and Australia were observed in three articles each.

**Table 3:** Collaboration patterns of the top 50 most-cited articles

Type of collaboration	No. of articles	
Individual institutions	24	
Multi-institutional collaboration	13	
International collaborations	13	
	UK	5
	Ireland	4
	USA	3
	Australia	3
	Norway	2
	UAE	2
	Malaysia	2
	Denmark	2
	Germany	2
	Italy	2
	Jordan	2

### Keywords

An analysis of the 50 most-cited articles revealed a total of 96 distinct keywords. The five most frequently used keywords, which appeared at least 9 times, and their occurrence counts across the articles are presented in Table 4. The keyword analysis of the top 50 most-cited articles revealed that “pulpotomy” was the most frequently used term, appearing 29 times (Table 4). This was followed by “pulpitis” with 21 occurrences and “irreversible pulpitis” with 14 (Table 4). Other commonly used keywords included mineral trioxide aggregate, which appeared 10 times, and vital pulp therapy, which was used 9 times (Table 4).

**Table 4:** The top 5 most frequently used keywords in the most-cited articles

S. No.	Keywords	Frequency
1	Pulpotomy	29
2	Pulpitis	21
3	Irreversible pulpitis	14
4	Mineral trioxide aggregate	10
5	Vital pulp therapy	9

### Methodological design

From a methodological perspective, the majority of the top 50 most-cited articles were clinical studies. Notably, randomized clinical trials constituted the largest proportion, accounting for 37.7% of the publications, followed by non-randomized clinical trials (17%) and observational studies (11.4%) (Table 5). Review articles represented 7.6% (*n* = 4) of the sample, while basic science studies and clinical guidelines were each represented at a rate of 5.7% (*n* = 3) (Table 5). Systematic reviews accounted for 3.8% (*n* = 2), and umbrella reviews for 1.9% (*n* = 1), indicating a comparatively lower representation among the methodological categories (Table 5).

**Table 5:** Categorization of the first 50 articles by methodological design

Methodological design	No. of articles (percentage)
Randomized clinical trial	20 (37.7%)
Non-randomized clinical trial	9 (17%)
Observational study	6 (11.4%)
Systematic review and meta-analysis	5 (9.4%)
Review article	4 (7.6%)
Basic science	3 (5.7%)
Guideline	3 (5.7%)
Systematic review	2 (3.8%)
Umbrella review	1(1.9%)

## Discussion

VPT have increasingly gained clinical and scientific attention in recent years for the treatment of teeth with IP, as they introduce an alternative approach to cases that were conventionally managed entirely with RCT [19]. A growing number of high-quality randomized controlled trials and systematic reviews in this field have contributed to the evidence supporting the practicality and effectiveness of VPT [5, 19, 21, 39]. This interest is clearly reflected in the growing number of publications and the rising citation rates on the topic in recent years. Therefore, a bibliometric analysis of this topic is both timely and valuable in terms of its contribution to the field. In this study, various bibliometric methods were used to evaluate the main trends, prominent researchers, and collaborations in the literature, while VOSviewer software was employed for visual analyses [40, 41]. This software has similarly been used in various studies within the field of dentistry to analyze keyword co-occurrences [36], and co-authorship networks [29].

In recent years, there has been a noticeable increase in the literature on VPT in teeth with IP [3–5, 21]. The fact that the majority of the top 50 most cited articles analyzed in this study were published after 2015 indicates a growing scientific interest and influence in this field over the past decade. In this regard, our findings are consistent with the results of bibliometric studies conducted in other emerging research areas such as regenerative endodontics and micro-CT [28, 29, 36]. The relatively low citation counts of articles published in 2024 and 2025 [4, 19] may be due to the fact that these studies have not yet been widely recognized by researchers [42]. It takes time for articles to gather citations, so these recent studies will likely receive more recognition in the next few years [42]. In addition, the ‘snowball effect,’ whereby older publications gain progressively more citations over time as their visibility increases independently of content and quality, may partly account for the higher citation rates observed in earlier studies [43]. Both 2019 and 2022 were the most productive years, each with seven published articles. During the COVID-19 pandemic, which began in 2019, the need to shorten procedure times and enhance infection control may have contributed to an increased interest in minimally invasive procedures [44]. In this context, VPT has been suggested as an emergency treatment option for IP cases [44, 45], which may have contributed to the rise in publication numbers. The growing acceptance of more conservative approaches, such as pulpotomy, in recently updated position statements may also have contributed to this trend [8, 9, 46]. This indicates that the view that RCT is not always necessary in cases of IP may be becoming more widely recognized [8, 9].

The highest cited countries in this study, based on the total citation numbers, were Iran (*n* = 10, cit. = 805), followed by the USA (*n* = 7, cit. = 467), India (*n* = 6, cit. = 278), Jordan (*n* = 6, cit. = 556), and Ireland (*n* = 5, cit. = 679). In this analysis, Iran's dominant publication profile is consistent with the country's centralized research structures, widespread access to resources, and increasing trend in academic production in biomedical sciences [47, 48]. When the most cited institutions and authors in this field are examined, a correlation with their countries becomes apparent. Among the most cited studies, Asgary S from Shahid Beheshti University in Iran (*n* = 9, cit. = 726; Asgary S *n* = 7, cit. = 600), Taha NA from Jordan University of Science and Technology (*n* = 6, cit. = 556; Taha NA *n* = 6, cit. = 556), and Duncan HF from Dublin Dental University Hospital in Ireland (*n* = 5, cit. = 679; Duncan HF *n* = 4, cit. = 624) clearly stand out. The most cited articles likely share the common trait of having a first author with well-established and recognized expertise in the field [49]. Asgary et al. have demonstrated the success of VPT in managing IP cases through their research. Their multicenter randomized controlled trial, in particular, showed that pulpotomy offers long-term outcomes in permanent teeth comparable to RCT [3]. Asgary et al. have also carried out extensive studies on key biomaterials like MTA, which are important for the development of VPT [50]. Similarly, Taha et al. have reported through their clinical studies on symptomatic IP cases that pulpotomy may be an effective alternative to RCT and that this approach can also improve patient comfort [5, 39]. Moreover, the highly cited position statement published in 2019 with Duncan HF as the first author, along with his comprehensive single-author review published in 2022, has provided a foundation for numerous studies and significantly contributed to the advancement of VPT [14, 46].

In terms of collaboration patterns, the findings of the present study indicate that although a substantial proportion of highly cited publications were produced as single-institution studies, international and multi-institutional collaborations constitute a considerable share of influential publications in the field. International collaborations were most frequently conducted with the United Kingdom, followed by Ireland, the United States, and Australia. This trend may be related to the greater allocation of resources to research and publication activities in economically stronger countries [51].

In this bibliometric study, the majority of the 50 most-cited articles were published in leading journals in the field of endodontics. Notably, the IEJ, accounting for 32% of the publications, and the JOE, with a 20% share, together comprise more than half of the articles on the list. In this respect, our study aligns with previous bibliometric studies conducted in the field of endodontics [28, 52]. This highlights the scientific influence of these two journals within the endodontic literature[28, 52]. At the same time, it is not only the numerical prominence of these two journals that stands out, but also their publication of clinical studies on VPT in patients diagnosed with IP, as well as influential guidelines and reviews, which highlights their potential to shape scientific direction in the field [4, 5, 9, 19, 46]. Considering that the 2024 impact factors of the journals in question are 3.6 and 7.1, it can be concluded that publications in high-impact journals offer a notable advantage in terms of both scientific visibility and citation frequency [42]. On the other hand, the relatively low number of articles in multidisciplinary journals may suggest that research on VPT in IP cases is still mostly shaped by specialists and has not yet gained sufficient attention in the wider scientific community. Supporting this, a survey study showed that specialists prefer pulpotomy more than general dentists in the treatment of IP [53].

The keywords from the 50 most-cited articles were analyzed to reveal the main trends and focal areas within the literature [29]. Due to the absence of keyword information in some of the articles listed in Table S2, these articles were not included in the analysis (23, 29, 33, 37, 43, 46, 49). As expected, the prominence of terms such as “pulpotomy,” “pulpitis,” and “irreversible pulpitis” among the most frequently used keywords indicates that research is largely shaped around diagnosis-based clinical approaches and treatment methods. At the same time, the prominence of biomaterial-related keywords like 'mineral trioxide aggregate' highlights the growing visibility of material-focused research within the field [35]. However, in our study, terms related to patient satisfaction, postoperative pain, or long-term success rates were limited among the keywords. Therefore, in literature searches focusing on VPT in patients with IP, it is important to include concepts related to patient satisfaction and clinical outcomes in keyword strategies, rather than limiting the search to procedural or material terms. In addition, interest in pulpal healing biomarkers [54], regenerative approaches [55], and artificial intelligence–based [56] studies is gradually increasing; these topics are expected to receive more attention in future research related to vital pulp therapy.

In this study, most of the highly cited publications were randomized clinical trials, accounting for 37.7% (*n* = 20, 1412 citations), and non-randomized clinical studies, accounting for 17% (*n* = 9, 667 citations). This suggests that research on VPT is largely conducted at the clinical level with a strong emphasis on patient outcomes [5, 11]. The prominence of clinical studies reflects the necessity of evaluating the efficacy and safety of proposed treatment approaches [57]. However, randomized controlled trials, which are positioned at the top of the evidence hierarchy [57], are more closely aligned with the publication priorities of high–impact factor journals, and this alignment is thought to influence the visibility and citation rates of such studies [58]. These findings are in line with other bibliometric studies in dentistry regarding the most cited articles [59, 60]. However, previous studies on novel treatment methods mostly focused on in vitro research [28–30]. The focus on clinical studies in our review may be due to the fact that IP is generally diagnosed based on clinical findings [1, 4], which likely excluded in vitro studies such as cell culture and animal studies. According to our analysis, systematic review articles accounted for 3.8% (*n* = 2204), systematic reviews with meta-analyses for 9.4% (*n* = 5652), and umbrella review articles for 1.9% (n = 144). Although the level of evidence is high, these proportions may remain low because the field is still developing clinically, and the number of studies suitable for review articles is not yet sufficient [42]. Although guidelines represent only 5.7% of the publications, the fact that they received a high number of citations (*n* = 3, 574) is remarkable. This indicates that such sources are frequently cited in clinical decision-making processes and have a significant impact on the field [57].

This study has several limitations. First, only the WoS and Scopus databases were used. Since other sources such as PubMed, Google Scholar or Embase were not included, some important publications indexed in those databases may have been excluded from the study. In addition, only articles published in English were evaluated, while studies in other languages were excluded, which may have introduced language bias. Co-citation and bibliographic coupling analyses were not performed in this study. Self-citations were not separated from the total citation counts; therefore, the citation impact of some articles may appear higher than it actually is due to author- or journal-level self-citation practices. Citation counts alone may not fully reflect the scientific value of a study. As this study focused only on the top 50 most-cited articles, some newer and potentially important works that could influence current research priorities may not be included. The analyses were based solely on the institutional affiliation of the first author, which may have led to an underrepresentation of the institutions or countries of other researchers involved in the same studies. In addition, collaboration networks were not analyzed in detail, which may limit the interpretation of country or institution-based rankings. Furthermore, this study considered only the methodological design and did not assess the methodological quality of the articles. Therefore, while the findings provide a general overview of the structure of the literature, they do not offer a direct evaluation of the scientific quality of the included articles.

This study demonstrates a major strength through the systematic and detailed bibliometric evaluation of the most influential publications on vital pulp therapy in irreversible pulpitis. From a clinical perspective, the findings may contribute to evidence-based clinical decision-making and reflect the growing adoption of pulp-preserving approaches.

## Conclusion

The majority of the most cited articles were published after 2015, indicating a rapidly growing scientific interest in the field in recent years. Publications were largely concentrated in high-impact journals such as the IEJ and the JOE. Asgary S was the author with the highest number of publications, while Shahid Beheshti University in Iran was the institution with the greatest contribution. Most of the publications were clinical studies; however, the number of systematic reviews and meta-analyses was more limited. Therefore, there remains a need for high-level evidence studies and multicenter research collaborations to further deepen the knowledge base in the field.

This bibliometric analysis demonstrates a growing scientific interest in VPT for IP, with publications concentrated in a limited number of countries, institutions, and high-impact journals. The observed trends reflect the increasing acceptance of minimally invasive, pulp-preserving approaches, particularly pulpotomy, in clinical practice and guideline development. Nevertheless, important gaps remain. Future research should focus on the development of multicenter registries, the standardization of outcome reporting, and the improvement of data-sharing practices. In addition, high-level comparative studies are needed to evaluate differences between pediatric and adult populations and to directly compare the outcomes of pulpotomy and full pulpectomy. These efforts are expected to contribute directly to the development of future clinical protocols and the refinement of evidence-based guidelines in endodontics.

## Supplementary Information

Below is the link to the electronic supplementary material.

Supplementary file1 (DOCX 12 kb)

Supplementary file2 (DOCX 20 kb)

## Data Availability

The datasets used and/or analysed during the current study available from the corresponding author on reasonable request.
