# Supplementary material for: Top-cited articles on vital pulp therapy for irreversible pulpitis in permanent teeth: a bibliometric analysis
Source: Odontology. 2026 Jan 28;114(4):2626–36. doi: 10.1007/s10266-026-01327-1 (PMC13630917; doi:10.1007/s10266-026-01327-1)
Supplement: Supplementary file 1 [file 10266_2026_1327_MOESM1_ESM.docx]

Table S1 Search Terms Used in the Study

| #1 | TS=((permanent OR secondary OR adult ) AND (dentition* OR t??th)) |
| --- | --- |
| #2 | TS=(pulpotom* OR partial pulpotomy OR miniature pulpotomy OR total pulpotomy OR Cvek pulpotomy OR shallow pulpotomy OR pulp amputation OR direct pulp capping OR indirect pulp capping OR (vital pulp AND (treatment OR therapy))) |
| #3 | TS=(((irreversible OR symptomatic OR acute OR asymptomatic OR chronic OR hyperplastic) AND pulpitis) OR pulpal inflammation) |
| #4 | #1 AND #2 AND #3 |
