# Supplementary material for: Top-cited articles on vital pulp therapy for irreversible pulpitis in permanent teeth: a bibliometric analysis
Source: Odontology. 2026 Jan 28;114(4):2626–36. doi: 10.1007/s10266-026-01327-1 (PMC13630917; doi:10.1007/s10266-026-01327-1)
Supplement: Supplementary file 2 [file 10266_2026_1327_MOESM2_ESM.docx]

Table S2 List of the 50 most-cited articles

| **Rank** | **Title of the article** | **No. of citation**  **(Web of Science)** | | **No. of citation**  **Scopus** |
| --- | --- | --- | --- | --- |
|  |  | **Total** | **Avg. per year** |  |
| 1 | European Society of Endodontology (ESE) developed by:, Duncan HF, Galler KM, Tomson PL, Simon S, El‐Karim I, Bjørndal L (2019) European Society of Endodontology position statement: Management of deep caries and the exposed pulp. International Endodontic Journal 52(7), 923–934. | 356 | 59.33 | 399 |
| 2 | Taha NA, Khazali MA (2017) Partial pulpotomy in mature permanent teeth with clinical signs indicative of irreversible pulpitis: a randomized clinical trial. Journal of Endodontics 43(9), 1417–1421. | 155 | 19.38 | 166 |
| 3 | Witherspoon DE, Small JC, Harris GZ (2006) Mineral trioxide aggregate pulpotomies: a case series outcomes assessment. The Journal of the American Dental Association 137(5), 610–618. | 145 | 7.63 | 148 |
| 4 | Cushley S, Duncan HF, Lappin MJ, Tomson PL, Lundy FT, Cooper P, El Karim IA (2019) Pulpotomy for mature carious teeth with symptoms of irreversible pulpitis: a systematic review. Journal of Dentistry 88, 103158. | 136 | 22.67 | 155 |
| 5 | Duncan HF (2022) Present status and future directions—vital pulp treatment and pulp preservation strategies. International Endodontic Journal 55, 497–511. | 128 | 42.67 | 146 |
| 6 | Linsuwanont P, Wimonsutthikul K, Pothimoke U, Santiwong B (2017) Treatment outcomes of mineral trioxide aggregate pulpotomy in vital permanent teeth with carious pulp exposure: the retrospective study. Journal of Endodontics 43(2), 225–230. | 116 | 14.5 | 110 |
| 7 | Taha NA, Abdelkhader SZ (2018) Outcome of full pulpotomy using Biodentine in adult patients with symptoms indicative of irreversible pulpitis. International Endodontic Journal 51(8), 819–828. | 116 | 16.57 | 122 |
| 8 | Asgary S, Eghbal MJ, Fazlyab M, Baghban AA, Ghoddusi J (2015) Five-year results of vital pulp therapy in permanent molars with irreversible pulpitis: a non-inferiority multicenter randomized clinical trial. Clinical Oral Investigations 19(2), 335–341. | 114 | 11.4 | 150 |
| 9 | Taha NA, Ahmad MB, Ghanim AJ (2017) Assessment of mineral trioxide aggregate pulpotomy in mature permanent teeth with carious exposures. International Endodontic Journal 50(2), 117–125. | 114 | 14.25 | 134 |
| 10 | Alqaderi H, Lee CT, Borzangy S, Pagonis TC (2016) Coronal pulpotomy for cariously exposed permanent posterior teeth with closed apices: a systematic review and meta-analysis. Journal of Dentistry 44, 1–7. | 105 | 11.67 | 115 |
| 11 | Lin LM, Ricucci D, Saoud TM, Sigurdsson A, Kahler B (2020) Vital pulp therapy of mature permanent teeth with irreversible pulpitis from the perspective of pulp biology. Australian Endodontic Journal 46(1), 154–166. | 101 | 20.2 | 115 |
| 12 | Asgary S, Hassanizadeh R, Torabzadeh H, Eghbal MJ (2018) Treatment outcomes of 4 vital pulp therapies in mature molars. Journal of Endodontics 44(4), 529–535. | 96 | 13.71 | 97 |
| 13 | Asgary S, Eghbal MJ, Ghoddusi J (2014) Two-year results of vital pulp therapy in permanent molars with irreversible pulpitis: an ongoing multicenter randomized clinical trial. Clinical Oral Investigations 18(2), 635–641. | 94 | 8.54 | 89 |
| 14 | Qudeimat MA, Alyahya A, Hasan AA, Barrieshi‐Nusair KM (2017) Mineral trioxide aggregate pulpotomy for permanent molars with clinical signs indicative of irreversible pulpitis: a preliminary study. International Endodontic Journal 50(2), 126–134. | 93 | 11.62 | 104 |
| 15 | Eghbal MJ, Asgary S, Baglue RA, Parirokh M, Ghoddusi J (2009) MTA pulpotomy of human permanent molars with irreversible pulpitis. Australian Endodontic Journal 35(1), 4–8. | 92 | 5.41 | 122 |
| 16 | Galani M, Tewari S, Sangwan P, Mittal S, Kumar V, Duhan J (2017) Comparative evaluation of postoperative pain and success rate after pulpotomy and root canal treatment in cariously exposed mature permanent molars: a randomized controlled trial. Journal of Endodontics 43(12), 1953–1962. | 90 | 11.25 | 100 |
| 17 | Duncan HF, Kirkevang LL, Peters OA, El‐Karim I, Krastl G, Del Fabbro M, Kopp I (2023) Treatment of pulpal and apical disease: the European Society of Endodontology (ESE) S3‐level clinical practice guideline. International Endodontic Journal 56, 238–295. | 90 | 45 | 93 |
| 18 | Parinyaprom N, Nirunsittirat A, Chuveera P, Lampang SN, Srisuwan T, Sastraruji T, Chompu-Inwai P (2018) Outcomes of direct pulp capping by using either ProRoot mineral trioxide aggregate or Biodentine in permanent teeth with carious pulp exposure in 6- to 18-year-old patients: a randomized controlled trial. Journal of Endodontics 44(3), 341–348. | 88 | 12.57 | 88 |
| 19 | Taha NA, Abdulkhader SZ (2018) Full pulpotomy with Biodentine in symptomatic young permanent teeth with carious exposure. Journal of Endodontics 44(6), 932–937. | 88 | 12.57 | 103 |
| 20 | Asgary S, Eghbal MJ (2013) Treatment outcomes of pulpotomy in permanent molars with irreversible pulpitis using biomaterials: a multi-center randomized controlled trial. Acta Odontologica Scandinavica 71(1), 130–136. | 85 | 6.54 | 108 |
| 21 | Elmsmari F, Ruiz XF, Miró Q, Feijoo-Pato N, Durán-Sindreu F, Olivieri JG (2019) Outcome of partial pulpotomy in cariously exposed posterior permanent teeth: a systematic review and meta-analysis. Journal of Endodontics 45(11), 1296–1306. | 85 | 14.17 | 90 |
| 22 | Uesrichai N, Nirunsittirat A, Chuveera P, Srisuwan T, Sastraruji T, Chompu‐Inwai P (2019) Partial pulpotomy with two bioactive cements in permanent teeth of 6‐ to 18‐year‐old patients with signs and symptoms indicative of irreversible pulpitis: a noninferiority randomized controlled trial. International Endodontic Journal 52(6), 749–759. | 83 | 13.83 | 85 |
| 23 | Nosrat A, Seifi A, Asgary S (2013) Pulpotomy in caries‐exposed immature permanent molars using calcium‐enriched mixture cement or mineral trioxide aggregate: a randomized clinical trial. International Journal of Paediatric Dentistry 23(1), 56–63. | 79 | 6.08 | 102 |
| 24 | Asgary S, Eghbal MJ, Ghoddusi J, Yazdani S (2013) One-year results of vital pulp therapy in permanent molars with irreversible pulpitis: an ongoing multicenter, randomized, non-inferiority clinical trial. Clinical Oral Investigations 17(2), 431–439. | 79 | 6.08 | 95 |
| 25 | Li Y, Sui B, Dahl C, Bergeron B, Shipman P, Niu L, Tay FR (2019) Pulpotomy for carious pulp exposures in permanent teeth: a systematic review and meta-analysis. Journal of Dentistry 84, 1–8. | 76 | 12.67 | 78 |
| 26 | Asgary S, Eghbal MJ (2010) The effect of pulpotomy using a calcium-enriched mixture cement versus one-visit root canal therapy on postoperative pain relief in irreversible pulpitis: a randomized clinical trial. Odontology 98(2), 126–133. | 68 | 4.53 | 87 |
| 27 | Zanini M, Hennequin M, Cousson PY (2016) A review of criteria for the evaluation of pulpotomy outcomes in mature permanent teeth. Journal of Endodontics 42(8), 1167–1174. | 68 | 7.56 | 72 |
| 28 | Santos JM, Pereira JF, Marques A, Sequeira DB, Friedman S (2021) Vital pulp therapy in permanent mature posterior teeth with symptomatic irreversible pulpitis: a systematic review of treatment outcomes. Medicina 57(6), 573. | 68 | 17.00 | 66 |
| 29 | Asgary S, Eghbal MJ, Bagheban AA (2017) Long-term outcomes of pulpotomy in permanent teeth with irreversible pulpitis: a multi-center randomized controlled trial. American Journal of Dentistry 30(3), 151–155. | 64 | 8 | 72 |
| 30 | Careddu R, Duncan HF (2021) A prospective clinical study investigating the effectiveness of partial pulpotomy after relating preoperative symptoms to a new and established classification of pulpitis. International Endodontic Journal 54(12), 2156–2172. | 55 | 13.75 | 63 |
| 31 | Duncan HF, El‐Karim I, Dummer PM, Whitworth J, Nagendrababu V (2023) Factors that influence the outcome of pulpotomy in permanent teeth. International Endodontic Journal 56, 62–81. | 50 | 25 | 54 |
| 32 | Taha NA, Al‐Rawash MH, Imran ZA (2022) Outcome of full pulpotomy in mature permanent molars using 3 calcium silicate‐based materials: a parallel, double blind, randomized controlled trial. International Endodontic Journal 55(5), 416–429. | 47 | 15.67 | 48 |
| 33 | Oguntebi BR, DeSchepper EJ, Taylor TS, White CL, Pink FE (1992) Postoperative pain incidence related to the type of emergency treatment of symptomatic pulpitis. Oral Surgery, Oral Medicine, Oral Pathology 73(4), 479–483. | 46 | 1.84 | 50 |
| 34 | Kumar V, Juneja R, Duhan J, Sangwan P, Tewari S (2016) Comparative evaluation of platelet-rich fibrin, mineral trioxide aggregate, and calcium hydroxide as pulpotomy agents in permanent molars with irreversible pulpitis: a randomized controlled trial. Contemporary Clinical Dentistry 7(4), 512–518. | 45 | 5 | 53 |
| 35 | Leong DJX, Yap AU (2021) Vital pulp therapy in carious pulp–exposed permanent teeth: an umbrella review. Clinical Oral Investigations 25(12), 6743–6756. | 44 | 11 | 53 |
| 36 | Eren B, Onay EO, Ungor M (2018) Assessment of alternative emergency treatments for symptomatic irreversible pulpitis: a randomized clinical trial. International Endodontic Journal 51, e227–e237. | 43 | 6.14 | 46 |
| 37 | McDougal RA, Delano EO, Caplan D, Sigurdsson A, Trope M (2004) Success of an alternative for interim management of irreversible pulpitis. The Journal of the American Dental Association 135(12), 1707–1712. | 42 | 2.10 | 38 |
| 38 | Sharma R, Kumar V, Logani A, Chawla A, Mir RA, Sharma S, Kalaivani M (2021) Association between concentration of active MMP‐9 in pulpal blood and pulpotomy outcome in permanent mature teeth with irreversible pulpitis – a preliminary study. International Endodontic Journal 54(4), 479–489. | 37 | 9.25 | 34 |
| 39 | Jassal A, Nawal RR, Yadav S, Talwar S, Yadav S, Duncan HF (2023) Outcome of partial and full pulpotomy in cariously exposed mature molars with symptoms indicative of irreversible pulpitis: a randomized controlled trial. International Endodontic Journal 56(3), 331–344. | 37 | 18.5 | 39 |
| 40 | Caliskan MK (1993) Success of pulpotomy in the management of hyperplastic pulpitis. International Endodontic Journal 26(2), 142–148. | 36 | 1.5 | 41 |
| 41 | Ramani A, Sangwan P, Tewari S, Duhan J, Mittal S, Kumar V (2022) Comparative evaluation of complete and partial pulpotomy in mature permanent teeth with symptomatic irreversible pulpitis: a randomized clinical trial. International Endodontic Journal 55(5), 430–440. | 36 | 12 | 40 |
| 42 | Taha NA, Al-Khatib H (2022) 4-year follow-up of full pulpotomy in symptomatic mature permanent teeth with carious pulp exposure using a stainproof calcium silicate–based material. Journal of Endodontics 48(1), 87–95. | 36 | 12 | 31 |
| 43 | Eghbal MJ, Haeri A, Shahravan A, Kazemi A, Moazami F, Mozayeni MA, Asgary S (2020) Postendodontic pain after pulpotomy or root canal treatment in mature teeth with carious pulp exposure: a multicenter randomized controlled trial. Pain Research and Management 2020(1), 5853412. | 34 | 6.8 | 43 |
| 44 | Koli B, Chawla A, Logani A, Kumar V, Sharma S (2021) Combination of nonsurgical endodontic and vital pulp therapy for management of mature permanent mandibular molar teeth with symptomatic irreversible pulpitis and apical periodontitis. Journal of Endodontics 47(3), 374–381. | 33 | 8.25 | 29 |
| 45 | Tomson PL, Vilela Bastos J, Jacimovic J, Jakovljevic A, Pulikkotil SJ, Nagendrababu V (2023) Effectiveness of pulpotomy compared with root canal treatment in managing non‑traumatic pulpitis associated with spontaneous pain: a systematic review and meta‑analysis. International Endodontic Journal 56, 355–369. | 32 | 16 | 28 |
| 46 | Chueh LH, Chiang CP (2010) Histology of irreversible pulpitis premolars treated with mineral trioxide aggregate pulpotomy. Operative Dentistry 35(3), 370–374. | 31 | 2.07 | 36 |
| 47 | Croft K, Kervanto‑Seppälä S, Stangvaltaite L, Kerosuo E (2019) Management of deep carious lesions and pulps exposed during carious tissue removal in adults: a questionnaire study among dentists in Finland. Clinical Oral Investigations 23(3), 1271–1280. | 29 | 4.83 | 30 |
| 48 | Yong D, Cathro P (2021) Conservative pulp therapy in the management of reversible and irreversible pulpitis. Australian Dental Journal 66, S4–S14. | 29 | 7.25 | 29 |
| 49 | Ather AM, Patel B, Gelfond JAL, Ruparel NB (2022) Outcome of pulpotomy in permanent teeth with irreversible pulpitis: a systematic review and meta-analysis. Scientific Reports 12(1), 19664. | 28 | 9.33 | 30 |
| 50 | Villat C, Grosgogeat B, Seux D, Farge P (2013) Conservative approach of a symptomatic carious immature permanent tooth using a tricalcium silicate cement (Biodentine): a case report. Restorative Dentistry & Endodontics 38(4), 258. | 27 | 2.08 | - |
